# Antibiotic Toxicity Profiles of *Escherichia coli* Strains Lacking DNA Methyltransferases

**DOI:** 10.1021/acsomega.1c00378

**Published:** 2021-03-15

**Authors:** Zheng Chen, Hailin Wang

**Affiliations:** † State Key Laboratory of Environmental Chemistry and Ecotoxicology, Research Center for Eco-Environmental Sciences, Chinese Academy of Sciences, Beijing 100085, China; ‡ University of Chinese Academy of Sciences, Beijing 100049, China; § Institute of Environment and Health, Jianghan University, Wuhan 430056, China

## Abstract

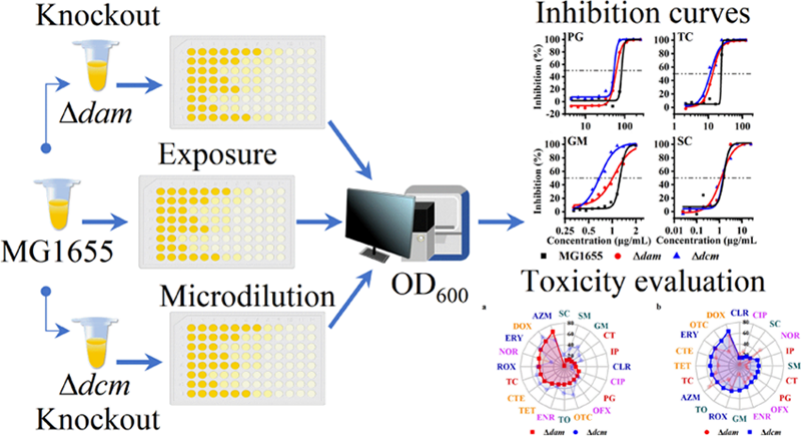

Antibiotic-resistant bacteria are causing more antibiotic treatment failures. Developing new antibiotics and identifying bacterial targets will help to mitigate the emergence and reduce the spread of antibiotic resistance in the environment. We investigated whether DNA methyltransferase (MTase) can be an adjunct target for improving antibiotic toxicity. We used *Escherichia coli* as an example. The genes encoding DNA adenine MTase and cytosine MTase, *dam* and *dcm*, respectively, were separately knocked out using the λRed system in *E. coli* MG1655. MG1655 and the two knockout strains were separately exposed in 96-well plates to 20 antibiotics from five classes. The EC_50_ values of almost all of the tested antibiotics were lower in the *dam* and *dcm* knockout lines than that of the control. Our statistical analysis showed that the variations observed in EC_50_ values were independent of the mechanism underlying each antibiotic’s mechanistic action.

## 1 Introduction

The growing issue of antibiotic resistance, especially in gram-negative bacteria, is threatening global health and food safety.^1^ Although antibiotic resistance occurs naturally,^2^ the misuse or overuse of antibiotics for human and veterinary health has exacerbated the emergence and spread of antibiotic resistance.^3−8^ From 2000 to 2015, the consumption rate of antibiotics increased by 39% around the world, reaching 42.3 billion defined daily doses.^9^ However, antibiotics for human and veterinary use are not well metabolized and up to 90% of that are released into the environment.^10,11^ As reported previously, many antibiotics were detected as an unchanged form in different water and soil environments.^12−15^ In China, due to the absence of effective removal techniques in sewage treatment plants, antibiotics such as tetracycline (TET), oxytetracycline (OTC), and ciprofloxacin (CIP) were detected at a concentration from a few ng/L to tens μg/L in both influents and effluents.^16^ Additionally, 94 antibiotics were detected in the surface water and the groundwater at median concentrations of up to 100 and 10 ng/L, respectively.^10^ In soil samples from China, 44 antibiotics from four classes were detected at a rate from 81 to 100%.^17^ In soil samples near the feedlots of China, the maximum concentrations of detected chlortetracycline (CTE) and OTC were 12.9 and 4.24 mg/kg, respectively.^18^ Antibiotics in the soil can transfer to plants and influence growth.^19^ Notably, in an environment with antibiotic residues, resistant bacteria will be selected, accumulated, and spread prior to nonresistant bacteria.^20^ Antibiotic-resistant bacteria and antibiotic-resistant genes can spread among different organisms and species in the environment,^21−26^ which will lead to more severe antibiotic resistance.

To mitigate the emergence and spread of antibiotic resistance, reduction in antibiotic use and improvement in antibiotic efficacy is crucial, which requires investigation of adjunct targets for antibiotics.^27^ Recently, DNA methyltransferase (MTase) has shown potential. In *Escherichia coli*, DNA adenine methyltransferase (Dam, EC 2.1.1.72) mediates the generation of N^6^-methyladenine (6mA), and DNA cytosine methyltransferase (Dcm, EC 2.1.1.37) mediates the generation of C^5^-methylcytosine (5mC) at the second C in the 5′-CC(A/T)GG-3′ motif.^28−32^ DNA methylation, one of the most important and widespread epigenetic modifications, was shown to be involved in bacterial survival during antibiotic exposure.^32−34^ Moreover, DNA methylation (6mA or 5mC) plays an important role in regulating many biological processes including restriction–modification systems, replication initiation, mismatch repair, virulence persistence, and global gene regulation.^35−41^

In view of the above, this paper aims to (1) investigate whether DNA methyltransferase (MTase) can be an adjunct target and (2) whether the antibiotic toxicity can be improved. Here, the genes encoding Dam and Dcm (*dam* and *dcm*, respectively) were knocked out in *E. coli* MG1655 using the λRed system. The microdilution method was then used to assess the exposure of 20 antibiotics from five classes (β-lactams, tetracyclines, quinolones, aminoglycosides, and macrolides) using 96-well plates. The inhibition rates of different antibiotics against each *E. coli* strain were then determined. The concentrations required for the 50% maximal effect (EC_50_) were determined to compare and assess the toxicity of each antibiotic against three different *E. coli* strains. The overlap of 95% confidence intervals was used to assess significant differences in the EC_50_ values. This investigation provides a new insight to reduce the entry of antibiotics into the environment from the source and to extend the service life of existing antibiotics.

## 2 Results and Discussion

### 2.1 Growth Curve Determination

Following the construction of the *dam* and *dcm* knockout strains, their growth curves were determined by OD_600_ measurements over 24 h at 37 °C, with MG1655 serving as the control. Both *dam* and *dcm* have been previously shown to be nonessential genes.^42^ The growth curves of MG1655, Δ*dam*, and Δ*dcm* showed no growth rate differences in our experiments (Figure S3a). This confirms that *dam* or *dcm* knockouts in MG1655 do not affect the growth rate under normal culture conditions, though 6mA and 5mC were demonstrated to be functional in regulating many biological processes. Therefore, we consider that Dam and Dcm are not proper targets of the antibacterial agents per se but are potential adjunct targets in the presence of antibiotics.

### 2.2 Bacterial Exposure to Solvents of Antibiotics

Before conducting the exposure experiments, we assessed the effects of the solvents on the growth of MG1655, Δ*dam*, and Δ*dcm*. As shown in Figure S3a, the growth of MG1655 is a continuous process, which means that the growth rate will not decrease or increase sharply and suddenly under stable culture conditions. Therefore, we assessed the effects by comparing the OD_600_ value of the negative control with those of the exposure groups after 12 h of incubation. As a result, MG1655, Δ*dam*, and Δ*dcm* did not show any obvious differences in the OD_600_ values under the exposure of 1% DMSO or 100 μM NaOH (Figure S3b–d).

### 2.3 Curve Fitting of Inhibition Rates

As discussed above, the various solvents used to dissolve the antibiotics and the gene knockouts (*dam* and *dcm*) were both confirmed to not influence the growth rate of MG1655, Δ*dam*, and Δ*dcm* (Figure S3). Therefore, we performed antibiotic exposure experiments wherein each strain was exposed to 11 concentrations of each antibiotic, and the OD_600_ values of the exposed samples were measured after 12 h of incubation when the bacteria were in the log phase.

**Figure 1:**
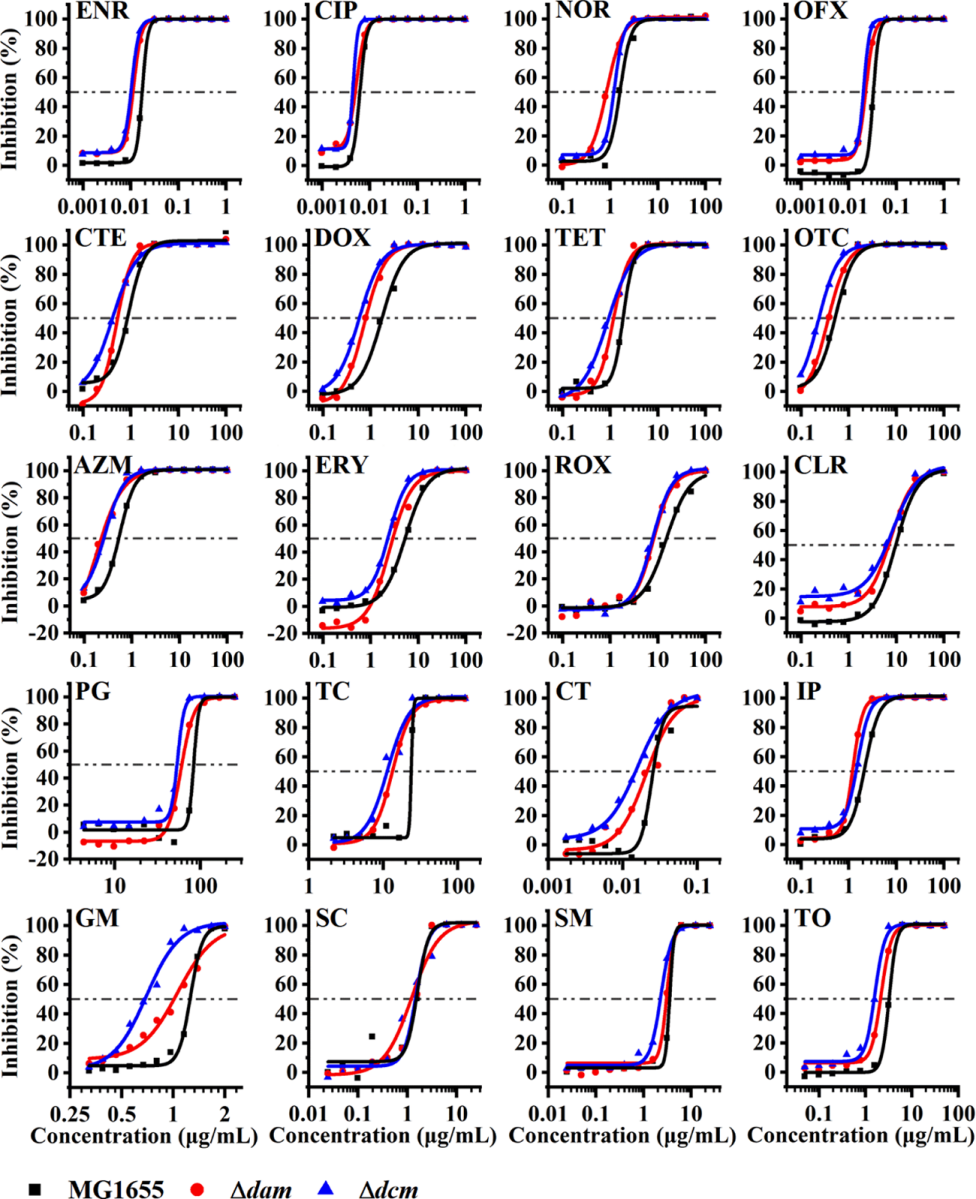
Inhibition curves of five classes of antibiotics against MG1655, Δ*dam*, and Δ*dcm*. Three strains were, respectively, exposed with β-lactams, aminoglycosides, tetracyclines, macrolides, and quinolones from the initial culture at 37 °C. The symbols represent the calculated inhibition rates at different antibiotic concentrations, and the lines represent inhibition curves that were nonlinearly fitted.

This enabled us to curve fit the inhibition rate of each antibiotic against the different strains using the software. As a result, the curve for the inhibition rate of a single exposure was well fitted by the logistic function and the Levenberg–Marquardt (LM) iterative algorithm (Figure 1). All 20 of the antibiotics from the five classes generated different dose-dependent inhibition effects against MG1655, Δ*dam*, and Δ*dcm* (Figure 1). Furthermore, Δ*dam* and Δ*dcm* required lower amounts of each antibiotic than the MG1655 control strain when the same inhibition rate of 50% was reached. Indeed, at the same concentrations, almost all of the tested antibiotics produced a higher inhibition rate in Δ*dam* and Δ*dcm* than in MG1655. However, what is noteworthy that erythromycin (ERY), CTE, and procaine penicillin (PG) promoted the growth of Δ*dam* at concentrations <0.5, <0.1, and <30 μg/mL, respectively, inducing the hormesis effect. This phenomenon does not appear in the exposure experiment against Δ*dcm* and MG1655. However, it can be concluded that knocking out *dam* or *dcm* improves the inhibition properties of almost all the antibiotics tested against *E. coli* MG1655.

### 2.4 Dam or Dcm Deficiency Improves Antibiotic Toxicity in *E. coli* MG1655

The toxicity of the antibiotics tested against MG1655, Δ*dam*, and Δ*dcm* was characterized by the EC_50_ values obtained in this work. The EC_50_ for each antibiotic was calculated at the same time as the curve fitting was performed. Contrasting with the minimum inhibitory concentration (MIC) determination method, this method can avoid the potential errors caused by visual inspection. We also applied the overlap of the 95% confidence interval (CI) to evaluate significant differences in the EC_50_ values for each antibiotic against the three strains.^43−45^

**Figure 2:**
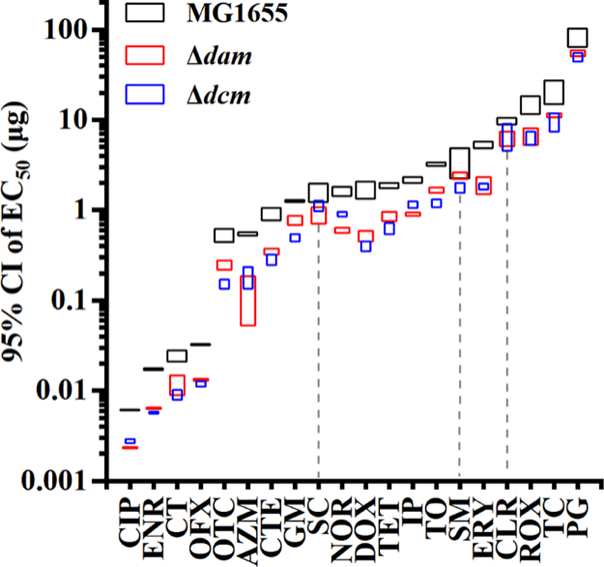
Significant difference analysis of EC_50_ of different antibiotics. Significant difference analysis of EC_50_ of different antibiotics against MG1655, Δ*dam*, and Δ*dcm* by the overlap of the 95% confidence interval. The dash lines indicate the overlap of the 95% confidence interval of Δ*dam* or Δ*dcm* with that of MG1655.

Our results showed that the 95% CI overlap for MG1655 was only observed for the exposure of streptomycin (SM) against Δ*dam* and the exposure of clarithromycin (CLR) and spectinomycin (SC) against Δ*dcm* (Figure 2). Thus, with the exception of the above three antibiotics, the EC_50_ values for each antibiotic against Δ*dam* and Δ*dcm* differed significantly from those against MG1655. However, the EC_50_ values of nine antibiotics (cefotaxime sodium (CT), azithromycin (AZM), SC, doxycycline (DOX), ERY, CLR, roxithromycin (ROX), ticarcillin sodium (TC), and PG) against Δ*dam* did not significantly differ from those against Δ*dcm* (Figure 2). Notably, none of the 95% CIs of the antibiotics tested against MG1655 overlapped with those against Δ*dam* and Δ*dcm* simultaneously (Figure 2). We then ranked the EC_50_ of each antibiotic against the three strains in ascending order (Table S6). The EC_50_ values for CIP, enrofloxacin (ENR), CT, and ofloxacin (OFX) are the minimal four of all of the antibiotics against MG1655, Δ*dam*, and Δ*dcm*. Furthermore, there are only slight variations in the EC_50_ ranking of each antibiotic against the different strains. In other words, the EC_50_ variation of a tested antibiotic is related to the absence of Dam or Dcm, but the effect is limited. Even so, according to the above discussion, we consider that Dam or Dcm deficiency improves the toxicity of antibiotics against *E. coli*.

### 2.5 Improvement of Toxicity was Independent of the Action Mechanism of Antibiotics

We performed a statistical analysis to investigate the observed variation in the EC_50_ values of the tested antibiotics and classified it according to their mechanisms of action. Quinolone antibiotics block DNA synthesis; aminoglycosides, tetracyclines, and macrolides inhibit bacterial protein synthesis; and β-lactams inhibit bacterial cell wall biosynthesis.^46−48^ We found that the EC_50_ values of the antibiotics with different modes of action against Δ*dam* or Δ*dcm* were lower than those against MG1655 (Figure S4a–c).

**Figure 3:**
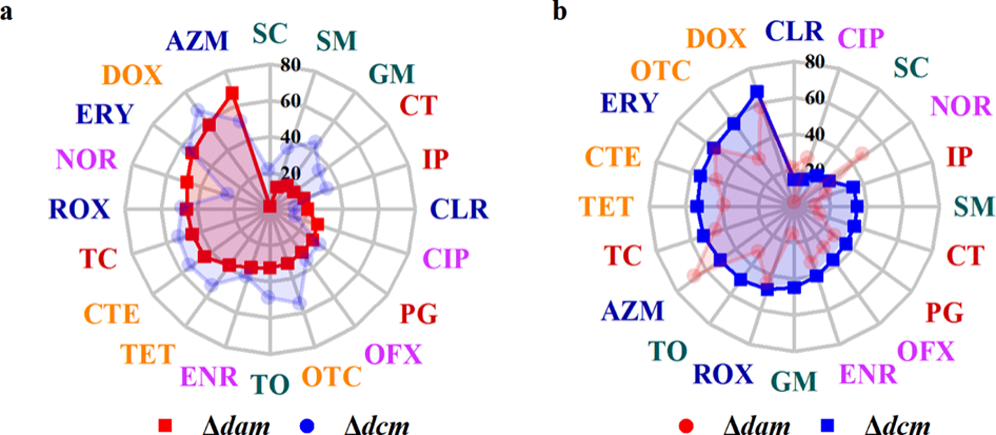
Relative EC_50_ reduction (%) of 20 tested antibiotics against Δ*dam* and Δ*dcm* with MG1655 as a control. (a) Relative EC_50_ reduction (%) of antibiotics against Δ*dam* in ascending order. Fonts of the same color represent the same class of antibiotics. (b) Relative EC_50_ reduction (%) of antibiotics against Δ*dcm* in ascending order. Fonts of the same color represent the same class of antibiotics.

Notably, the EC_50_ values of almost all of the antibiotics that inhibit bacterial protein synthesis were lower for the Δ*dcm* strain than for the Δ*dam* strain (Figure S4b). Thus, Dcm may be more important than Dam for bacterial survival during the exposure of antibiotics that inhibit bacterial protein synthesis. The reduction of EC_50_ induced by the deficiency of Dam or Dcm indicates the universality of DNA MTase as an adjunct target for improving the toxicity of antibiotics, and this is independent of the mode of action of the antibiotic. We then calculated the relative reduction in EC_50_ as a percentage, as shown in Figure 3. The EC_50_ reduction for AZM against Δ*dam* reached 67.4% (Figure 3a), a similar value (66.6%) to that of doxycycline against Δ*dcm* (Figure 3b). The reduction of EC_50_ values for tetracycline- and macrolide-type antibiotics was more significant than that of the other three classes. The reduced EC_50_ rate varies for diverse antibiotics against Δ*dam* or Δ*dcm*. This difference may be attributable to the different expression levels of the genes involved in bacterial survival, which is stimulated by different antibiotics.

As discussed above, under normal culture conditions, the absence of Dam or Dcm in the knockout lines did not reduce their growth rates. However, in the presence of antibiotics, 6mA-directed MMR in the Δ*dam* mutant would have created the inability to distinguish methylated and non-methylated sites, leading to toxic DNA breaks.^49^ In addition, the drug-induced error-prone Pol IV polymerase will cause an increased mutation rate, thereby exacerbating the emergence of DNA breaks and overwhelming the growth of bacteria cells.^50^ Besides, some important proteins such as ABC transporter involving in the transport of antibiotics were confirmed downregulated, which was induced by the deficiency of Dam.^34^ In contrast, Dcm is associated with drug resistance in *E. coli* by regulating the *SugE* gene expression during the stationary phase.^33^ However, in the log phase, the deficiency of Dcm may influence the expression of the transcription factor, which possibly results in some important genes involved in antibiotic resistance not being expressed as normal. The EC_50_ values of the tested antibiotics were thereby reduced by the deficiency of Dam or Dcm. Although the reduction in EC_50_ values is limited by the correlation mechanism, our results showed that the improvement of toxicity was independent of the action mechanism of antibiotics. Moreover, the random mutations of DNA, which are induced by the deficiency of Dam under the exposure of antibiotics, may help to provide a solution to antibiotic resistance mediated by antibiotic-resistant genes or plasmids.

## 3 Conclusions

In summary, we optimized the microdilution method to obtain a rapid evaluation of the toxicity of the tested antibiotics. We then performed exposure experiments using five antibiotic classes, from which we observed that the absence of Dam or Dcm caused the EC_50_ values of almost all of the tested antibiotics against MG1655 to reduce. This shows the universality and feasibility of DNA MTase as an adjunct target for improving the toxicity of antibiotics against *E. coli*. However, this effect is limited according to our result. The development of specific inhibitors targeting DNA MTase will be key to the application of this finding.

## 4 Experimental Section

### 4.1 Bacteria Strain and Agents

*E. coli* K-12 MG1655 strain (MG1655) was purchased from Tiandz Gene Technology (Beijing, China). Plasmids used for gene knockout were stored in our laboratory, including pKD46 (GenBank: AY048746.1) and pKD13 (GenBank: AY048744.1). l-Arabinose (CAS No. 5328-37-0, purity: >99%) was purchased from Solarbio (Beijing, China). The Q5 high-fidelity polymerase for polymerase chain reaction (PCR) was purchased from New England Biolabs (Ipswich, MA). Primers for the PCR were synthesized by Sangon Biotech (Shanghai, China). Lysogeny broth (LB) (5 g of yeast extract, 10 g of tryptone, and 10 g of sodium chloride per 1 L medium, pH 7.4) was applied to bacteria cultures. The LB solid medium (LB plate) was prepared by adding 2% agar (w/v) into the LB medium. Ampicillin (CAS No. 69-52-3, purity: USP Grade) and kanamycin (CAS No. 25389-94-0, purity: USP Grade) used for bacteria screening were purchased from Sangon Biotech, and the working concentrations were 100 and 20 μg/mL, respectively. Antibiotics used for exposure including β-lactams, tetracyclines, and quinolones were also purchased from Sangon Biotech. Other antibiotics including aminoglycosides and macrolides were purchased from Macklin (Shanghai, China). Dimethyl sulfoxide (DMSO, CAS No. 67-68-5, purity: >99.9%) was purchased from Beyotime (Shanghai, China). NaOH (CAS No. 1310-73-2, purity: >96%) and NaCl (CAS No. 7647-14-5, purity: >99.8%) were purchased from Sinopharm Chemical Reagent (Shanghai, China).

### 4.2 Constructing the *dam* and *dcm* Knockout Strains

The λRed knockout system was used to knock out *dam* and *dcm* genes as described previously.^51−53^ However, in this work, the lengths of the homologous arms of the substrate DNA were extended to 500 base pairs (bps) using the overlapping extension PCR (overlapping PCR).^54−56^ Briefly, the *kan* cassette, which encompasses a 500 bp DNA fragment upstream and downstream the target gene (*dam* or *dcm*) was PCR-generated using pKD13 and MG1655, respectively, as templates. After agarose gel purification, the three products were mixed at a mole ratio of 1:1:1 and used together as the template for the overlap PCR generating the substrate DNA. The gene knockout was further verified by the bacteria-broth PCR as well as Sanger sequencing. The elimination of pKD46 plasmid, which was temperature-sensitive, was accomplished in MG1655 by further bacterial culturing at 37 °C. The genotypes of the *dam* and *dcm* knockout strains were MG1655 Δ*dam*::*kan* (Δ*dam*) and MG1655 Δ*dcm*::*kan* (Δ*dcm*), respectively. All of the PCR primers were designed as shown in Figure S1, and their DNA sequences are listed in Tables S1–S3.

### 4.3 Determining OD_600_ Values and Growth Curves

OD_600_ measurements were determined on a Varioskan Flash microplate reader (Thermo, MA). Bacteria cells (200 μL/well) were cultured in flat-bottomed 96-well plates (3599, Corning, NY) at 37 °C and 200 rpm. OD_600_ was measured in triplicate every 2 h from the initial bacteria culture. Thus, the growth curves of the three strains were determined from the OD_600_ measurements over a 24 h period.^57,58^

### 4.4 Preparing Antibiotic Dilutions

The exposure experiments included five antibiotic classes: β-lactams (procaine penicillin, PG; ticarcillin sodium, TC; imipenem monohydrate, IP; and cefotaxime sodium, CT), tetracyclines (oxytetracycline hydrochloride, OTC; chlortetracycline hydrochloride, CTE; tetracycline, TET; and doxycycline, DOX), quinolones (norfloxacin, NOR; ciprofloxacin hydrochloride, CIP; enrofloxacin, ENR; and ofloxacin, OFX) aminoglycosides (tobramycin, TO; gentamicin, GM; streptomycin, SM; and spectinomycin, SC), and macrolides (roxithromycin, ROX; azithromycin, AZM; erythromycin, ERY; and clarithromycin, CLR), which were classified by their chemical structures. According to the standard from the Clinical and Laboratory Standards Institute (CLSI), 20 antibiotics were separately dissolved in the correct solvents at the proper concentrations (Table S4). Antibiotic dilutions were performed in 1 mL centrifuge tubes, and each antibiotic was successively diluted with the corresponding solvent to achieve 11 concentrations by the multiple dilution method.^59,60^ The initial dilution concentrations and ratios of the various antibiotics were pre-experimentally determined (Table S5). All of the dilutions were protected from light at 4 °C.

### 4.5 Preparing Bacterial Inoculums

Newly grown clones from the three strains (MG1655, Δ*dam*, and Δ*dcm*) were separately inoculated into 10 mL of fresh LB broth and incubated at 37 °C. When the OD_600_ reached ∼0.6, the bacteria broth was placed in ice for 15 min and then diluted with LB (1:10 000) to an OD_600_ of ∼0.01. The broth dilutions of MG1655, Δ*dam*, and Δ*dcm* strains were stored at 4 °C and then used as bacterial inoculums. All bacterial inoculums and antibiotic dilutions were prepared on a clean bench.

### 4.6 Bacterial Exposure to Solvents of Antibiotics

In this work, the solvents of antibiotic stock solutions were sterile water, dimethyl sulfoxide (DMSO), and 10 mM NaOH. The final concentration of the solvent in the antibiotic exposure tests was 1% for DMSO (v/v) or up to 100 μM for NaOH. MG1655, Δ*dam*, and Δ*dcm* from the initial bacteria culture were exposed in triplicate to 2 μL of DMSO or 10 mM NaOH, respectively, in 96-well plates, plus 198 μL of bacterial inoculum. The OD_600_ values of the samples were measured after 12 h of incubation.

### 4.7 Antibiotic Exposure Experiments

Dilutions of the 11 different antibiotic concentrations were added to wells in the same row of a 96-well plate. A 2 μL aliquot of the corresponding solvent was added to the first well of each row as a control. A 2 μL aliquot of antibiotic dilution was added to the wells from low to high concentration in the same row. Thus, 20 different antibiotics were added to three 96-well plates. Next, a 198 μL inoculum of a bacteria strain was carefully added to a well and mixed with the antibiotic dilution using a pipette (Figure S2). Altogether, 60 mL of each bacterial inoculum (strains MG1655, Δ*dam*, or Δ*dcm*) was prepared for the antibiotic exposure experiments. The Varioskan Flash microplate reader was used to measure the initial OD_600_ at 0 h of culturing the 96-well plate to which 200 μL/well of the samples was previously added. The plates were placed at 4 °C until all samples of the three strains had been added to the wells of 12 96-well plates. Every three 96-well plates containing the same strain were stacked together, fixed with rubber bands, and then carefully and smoothly placed in a constant temperature shaker. The three strains were then incubated at 37 °C and 200 rpm. OD_600_ was determined for each sample every 2 h over a 24 h period.

### 4.8 Curve Fitting of Inhibition Rates

The curve fitting of the inhibition rates was performed by OriginPro software (https://www.originlab.com/index.aspx?go=Products/Origin). First, the inhibition rates were calculated by taking the OD_600_ values at 12 h of culture as parameters (formula 1).^61,62^
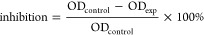
1
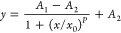
2
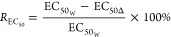
3OD_control_ refers to the OD_600_ of the control sample and OD_exp_ refers to the OD_600_ of the antibiotic-exposed sample. Second, nonlinear curve fitting of the inhibition rates was performed by the logistic function and the Levenberg–Marquardt (LM) iterative algorithm (formula 2).^63^ Third, the 50% maximal effect (EC_50_) of the various antibiotic concentrations against MG1655, Δ*dam*, and Δ*dcm* was calculated at the same time by curve fitting.^64^ A statistical analysis of the EC_50_ values was accomplished using the same software (formula 3). *R*_EC_50__ refers to the EC_50_ reduction rate of the test antibiotic, as induced by the *dam* or *dcm* knockout. EC_50_W__ is the EC_50_ value of an antibiotic targeting MG1655, whereas EC_50_Δ__ is the EC_50_ value of an antibiotic targeting Δ*dam* or Δ*dcm*.

## Supporting Information Available

Overlap PCR primers for the preparation of substrate DNA (*dam* knockout) (Table S1); overlap PCR primers for the preparation of substrate DNA (*dcm* knockout) (Table S2); primers for identification of *dam* and *dcm* knockouts using the bacteria PCR (Table S3); stock concentrations, solvents, and abbreviations of tested antibiotics (Table S4); dilution ratio and initial concentration of different antibiotics (Table S5); calculated EC_50_ values (μg/mL) of antibiotics against different strains (Table S6); schematic of the design of primers for the overlap PCR and the bacteria PCR (Figure S1); schematic of exposure experiments of antibiotics (Figure S2); evaluation of the growth rate after the gene knockout and under the exposure of different solvents of antibiotics (Figure S3); and EC_50_ values (μg/mL) of MG1655, Δ*dam*, and Δ*dcm* exposed to antibiotics of different action mechanisms (Figure S4) (PDF)
