# Supplementary material for: Antibiotic Toxicity Profiles of Escherichia coli Strains Lacking DNA Methyltransferases
Source: ACS Omega. 2021 Mar 15;6(11):7834–40. doi: 10.1021/acsomega.1c00378 (PMC7992158; doi:10.1021/acsomega.1c00378)
Supplement: Supplementary file 1 — ao1c00378_si_001.pdf [file ao1c00378_si_001.pdf]

Supporting Information:

Antibiotic Toxicity Profiles of *Escherichia coli* Strains Lacking DNA Methyltransferases

Zheng Chen[a, b] *and Hailin Wang*[a, b, c, *]

[a] *State Key Laboratory of Environmental Chemistry and Ecotoxicology, Research Center for Eco-Environmental Sciences, Chinese Academy of Sciences, Beijing, 100085, China;*

[b] *University of Chinese Academy of Sciences, Beijing, 100049, China;*

[c] *Institute of Environment and Health, Jianghan University, Wuhan, 430056, China*

**** Correspondence:***

Hailin Wang. E-mail: hlwang@rcees.ac.cn. Phone and Fax: +86-10-62849600

This Supporting Information contains 10 pages, 6 tables and 4 figures:

Table S1. Overlap PCR primers for the preparation of substrate DNA (*dam* knockout)

Table S2. Overlap PCR primers for the preparation of substrate DNA (*dcm* knockout)

Table S3. Primers for identification of *dam* and *dcm* knockouts using the bacteria PCR

Table S4. Stock concentrations, solvents, and abbreviations of tested antibiotics

Table S5. Dilution ratio and initial concentration of different antibiotics

Table S6. Calculated EC_{50} values (μg/mL) of antibiotics against different strains

Figure S1. Schematic of the design of primers for overlap PCR and bacteria PCR.

Figure S2. Schematic of exposure experiments of antibiotics.

Figure S3. Evaluation of growth rate after the gene knockout and under the exposure of different solvents of antibiotics.

Figure S4. EC_{50} values (μg/mL) of MG1655, Δ*dam*, and Δ*dcm* exposed to antibiotics of different action mechanisms.

Table S1. Overlap PCR primers for the preparation of substrate DNA (*dam* knockout)

Primer	DNA sequence (5′–3′)
PA1	ACTGAACCAGCTGCTCCT
PA2	CGACGGATCCCCGGAATTAATTCTGCTGACTAACTAATTACACCTTC
PA3	CAGCCTACACAATCGCTCAAGTTCTCAAGGAGAAGCGGATGAAACA
PA4	GACGTACTTCGCGCAGTTTA
PA5	AGAATTAATTCCGGGGATCCGTC
PA6	TCTTGAGCGATTGTGTAGGCTG

Table S2. Overlap PCR primers for the preparation of substrate DNA (*dcm* knockout)

Primer	DNA sequence (5′–3′)
PC1	CTCCGTGAAGAGTTCGAGCA
PC2	CGACGGATCCCCGGAATTAATTCTAGATTTCACCGGCCATCTGAG
PC3	CAGCCTACACAATCGCTCAAGATGGCCGACGTTCACGATAAG
PC4	GTAGTTGCGCCAGTTATTCAGGAC
PC5	CTCAGATGGCCGGTGAAATCTAGAATTAATTCCGGGGATCGTCG
PC6	CTTATCGTGAACGTCGGCCATCTTGAGCGATTGTGTAGGCTG

Table S3. Primers for identification of *dam* and *dcm* knockout using bacteria PCR

Primer	DNA sequence (5′–3′)
Fa	GTCGGAGCTTTCTCCACAGC
Ra	AATTGAGGGGGCAATCAAATAC
Fc	CGTATCACGCGTGGCAATCG
Rc	CGTGGCCCTAAATGGCTGTA
K2	CGGTGCCCTGAATGAACTGC
Kt	CGGCCACAGTCGATGAATCC

Table S4. Stock concentrations, solvents, and abbreviations of tested antibiotics

Drug class	Antibiotics	Abbreviation	CAS No.	Solvent	Concentration (mg/mL)
β-lactams	Procaine penicillin	PG	6130-64-9	DMSO	25
	Ticarcillin sodium	TC	4697-14-7	DMSO	25
	Imipenem monohydrate	IP	64221-86-9	DMSO	5
	Cefotaxime sodium	CT	64485-93-4	DMSO	25
Aminoglycosides	Tobramycin	TO	79645-27-5	Sterile water	5
	Gentamicin	GM	1405-41-0	Sterile water	20
	Streptomycin	SM	3810-74-0	Sterile water	5
	Spectinomycin	SC	22189-32-8	DMSO	2.5
Tetracyclines	Oxytetracycline hydrochloride	OTC	2058-46-0	DMSO	25
	Chlortetracycline hydrochloride	CTE	64-72-2	DMSO	10
	Tetracycline	TET	60-54-8	DMSO	10
	Doxycycline	DOX	24390-14-5	DMSO	10
Macrolides	Roxithromycin	ROX	80214-83-1	DMSO	25
	Azithromycin	AZM	83905-01-5	DMSO	25
	Erythromycin	ERY	114-07-8	DMSO	25
	Clarithromycin	CLR	81103-11-9	DMSO	10
Quinolones	Norfloxacin	NOR	70458-96-7	10mM NaOH	2
	Ciprofloxacin hydrochloride	CIP	93107-08-5	Sterile water	2
	Enrofloxacin	ENR	93106-60-6	10mM NaOH	2
	Ofloxacin	OFX	82419-36-1	10mM NaOH	2

Note: The stock concentrations, solvents, and abbreviations of antibiotics for exposure are optimized referring to Clinical and Laboratory Standards Institute (CLSI) standard.

Table S5. Dilution ratio and initial concentration of different antibiotics

Antibiotic	Dilution ratio	Initial concentration (μg/mL)	Antibiotic	Dilution ratio	Initial concentration (μg/mL)
PG	1.5	250	TET	2	100
TC	1.5	125	DOX	2	100
IP	2	100	ROX	2	100
CT	1.5	0.1	AZM	2	100
TO	2	50	ERY	2	100
GM	1.2	2	CLR	2	100
SM	2	25	NOR	2	100
SC	2	25	CIP	2	1
OTC	2	100	ENR	2	1
CTE	2	100	OFX	2	1

Table S6. Calculated EC_{50} values (μg/mL) of antibiotics against different strains

Antibiotic	MG1655	Er (±)	Δ*dam*	Er (±)	Δ*dcm*	Er (±)
CIP	0.00616	5E-5	0.00447	6.5E-5	0.0052	2.45E-4
ENR	0.01741	4.75E-4	0.01152	1.95E-4	0.0104	2.45E-4
CT	0.02454	0.00353	0.02057	0.00488	0.01596	0.00187
OFX	0.03252	7.9E-4	0.0229	5.2E-4	0.02071	0.00139
OTC	0.52892	0.08797	0.36236	0.03947	0.2305	0.02606
AZM	0.55063	0.02237	0.17955	0.09526	0.27287	0.06878
CTE	0.90956	0.14222	0.504	0.03594	0.41355	0.05001
GM	1.26289	0.03108	1.06019	0.11151	0.69718	0.05543
SC	1.60251	0.3712	1.21273	0.23397	1.5099	0.18403
NOR	1.61929	0.19326	0.8386	0.05323	1.2319	0.06086
DOX	1.7075	0.36761	0.73246	0.09145	0.57099	0.07076
TET	1.88252	0.1185	1.16247	0.12615	0.87413	0.11696
IP	2.17757	0.16012	1.22885	0.05439	1.54106	0.11951
TO	3.21888	0.12684	2.17946	0.14188	1.60692	0.1491
SM	3.56077	1.30201	3.1043	0.2464	2.32366	0.26839
ERY	5.30515	0.43814	2.49752	0.51902	2.39951	0.18101
CLR	9.65189	0.78924	7.66455	1.31444	8.22186	2.61797
ROX	14.86831	3.30321	8.04707	1.57254	7.70992	1.15877
TC	21.2519	6.25518	13.27311	0.60381	11.43135	2.57108
PG	83.34872	18.60187	59.23936	4.04209	54.01379	5.32648

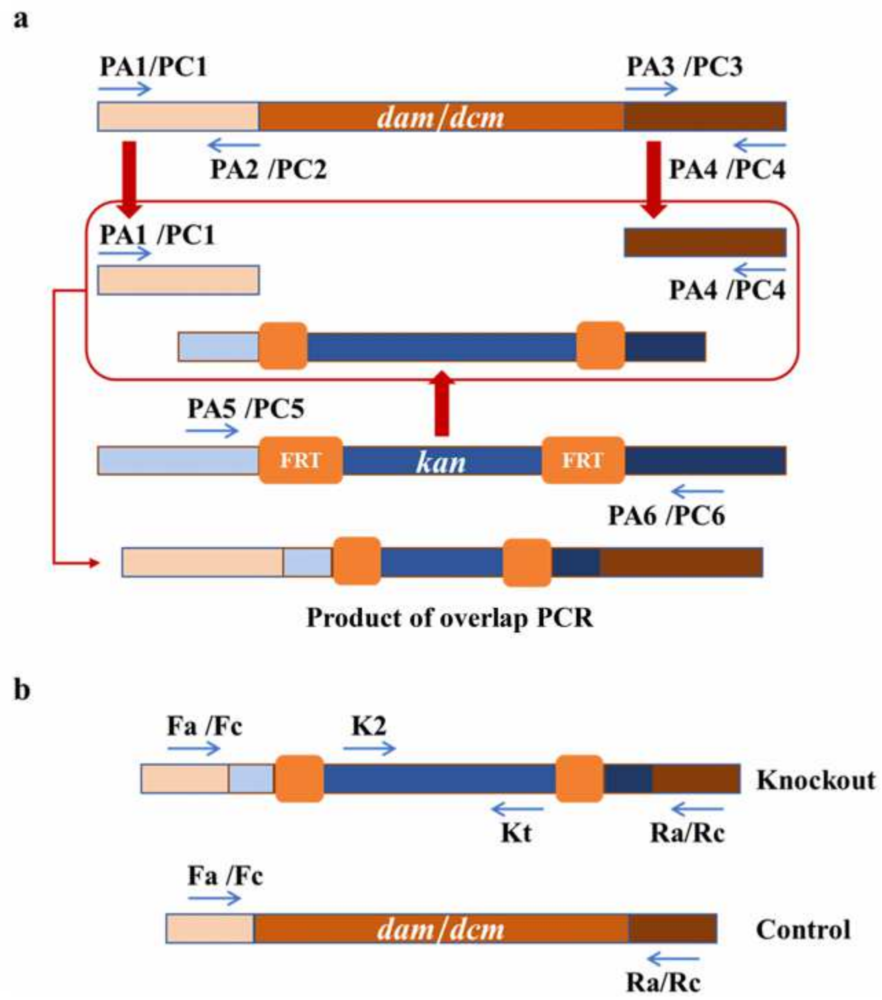

Figure S1. Schematic of the design of primers for overlap PCR and bacteria PCR.

a. The design of primers for overlap PCR aiming to prepare substrate DNA for the gene knockout of *dam* or *dcm*. **b.** The design of primers for bacteria PCR aiming to identify whether the gene knockout of *dam* or *dcm* is correct.

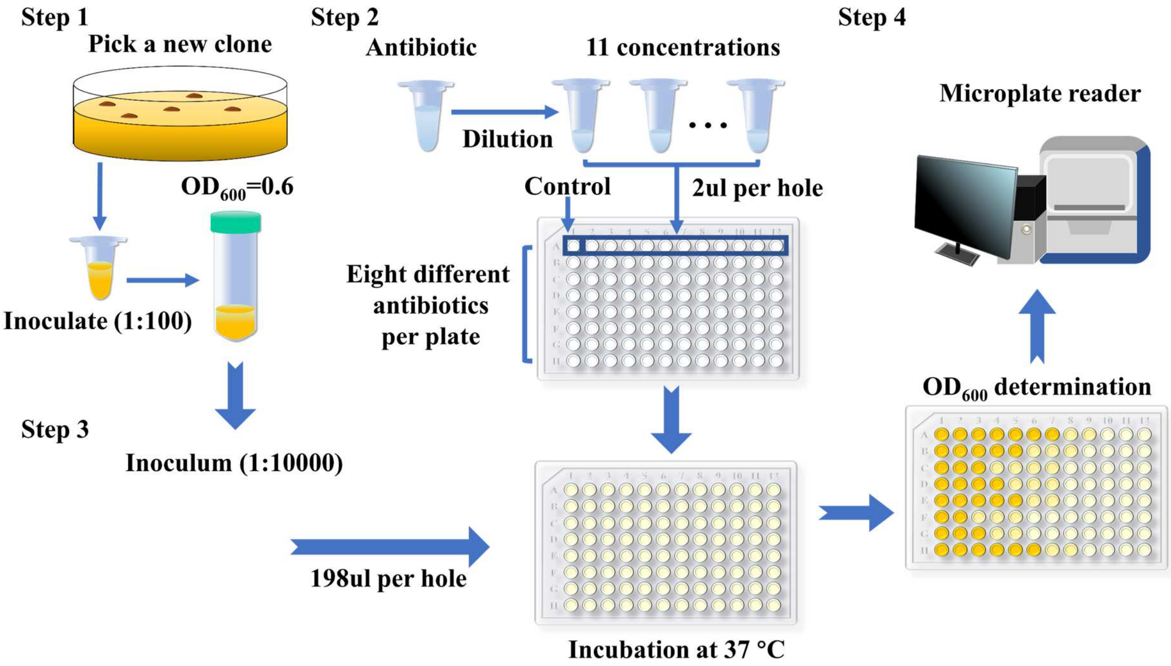

Figure S2. Schematic of exposure experiments of antibiotics. Step1: A new clone of a bacteria strain is picked and cultured to an OD_{600}=0.6 to prepare the bacteria inoculum. Step2: An antibiotic is diluted at a certain ratio and added to a 96-well plate. Step3: The diluted inoculum of a bacteria strain is added to the 96-well plate which contains different antibiotics. Step4: After 12 h of incubation at 37 °C, the 96-well plate is subject to a microplate reader to determine the OD_{600} of each exposure sample.

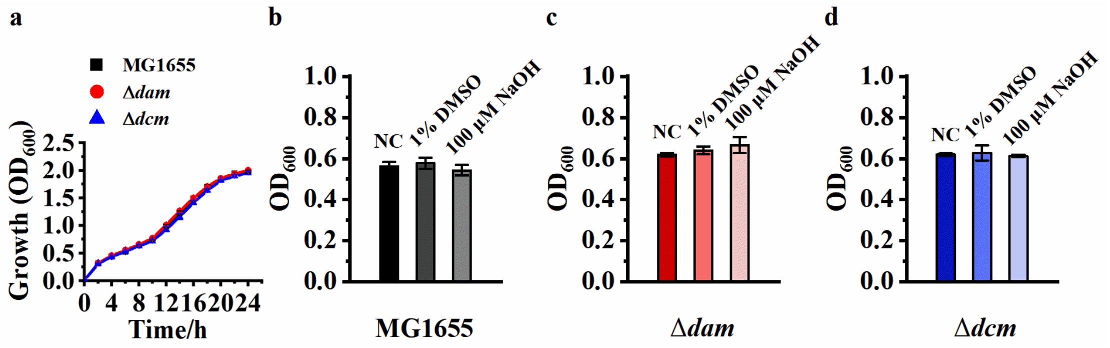

Figure S3. Evaluation of growth rate after the gene knockout and under the exposure of different solvents of antibiotics. a. The growth curves of MG1655, Δ*dam*, and Δ*dcm* over 24 hours of incubation at 37 °C. **(b, c, d).** The OD_{600} values of MG1655, Δ*dam*, and Δ*dcm* exposed to different solvents of antibiotics after 12 hours of incubation at 37 °C. Negative control (NC) indicates the bacteria strain was not exposed to any solvents of antibiotics.

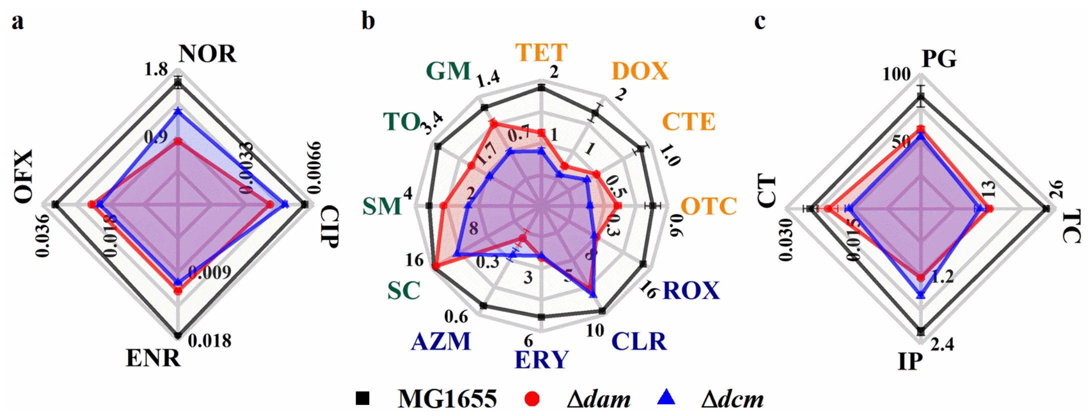

Figure S4. EC_{50} values (µg/mL) of MG1655, Δ*dam*, and Δ*dcm* exposed to antibiotics of different action mechanisms. a. EC_{50} values of antibiotics (quinolones) blocking DNA synthesis against MG1655, Δ*dam*, and Δ*dcm*. **b.** EC_{50} values of antibiotics (aminoglycosides, tetracyclines, and macrolides) inhibiting bacterial protein synthesis against MG1655, Δ*dam*, and Δ*dcm*. **c.** EC_{50} values of antibiotics (β-lactams) inhibiting bacterial cell wall biosynthesis against MG1655, Δ*dam*, and Δ*dcm*.
